# Air pollutants in bronchoalveolar lavage fluid and pulmonary tuberculosis: A mediation analysis of gene-specific methylation

**DOI:** 10.1016/j.isci.2023.108391

**Published:** 2023-11-03

**Authors:** Qiao Liu, Ye Ji, Li Wang, Zhongqi Li, Bilin Tao, Limei Zhu, Wei Lu, Leonardo Martinez, Yi Zeng, Jianming Wang

**Affiliations:** 1 Department of Epidemiology, Center for Global Health, School of Public Health, Nanjing Medical University, Nanjing 211166, P.R. China; 2 Department of Chronic Communicable Disease, Center for Disease Control and Prevention of Jiangsu Province, Nanjing 210009, P.R. China; 3 Department of Non-Communicable Disease, Center for Disease Control and Prevention of Jiangyin City, Wuxi 214434, P.R. China; 4 Department of Epidemiology, School of Public Health, Boston University, Boston, MA, USA; 5 Department of Tuberculosis, Nanjing Public Health Medical Center, Nanjing Second Hospital, Nanjing Hospital Affiliated to Nanjing University of Traditional Chinese Medicine, Nanjing 211113, P.R. China

**Keywords:** disease, environmental science, pollution

## Abstract

Particulate matter (PM) exposure could alter the risk of tuberculosis, but the underlying mechanism is still unclear. We enrolled 132 pulmonary tuberculosis (PTB) patients and 30 controls. Bronchoalveolar lavage fluid samples were collected from all participants to detect organochlorine pesticides, polycyclic aromatic hydrocarbons, metal elements, and DNA methylation of immunity-related genes. We observed that γ-HCH, Bap, Sr, Ag, and Sn were related to an increased risk of PTB, while Cu and Ba had a negative effect. IFN-γ, IL-17A, IL-2, and IL-23 had a higher level in the PTB group, while IL-4 was lower. The methylation of 18 CpG sites was statistically associated with PTB risk. The methylation at the IL-4_06_121 site showed a significant mediating role on γ-HCH, Sr, and Sn. Our study suggests that PM exposure can increase the risk of tuberculosis by affecting DNA methylation and cytokine expression.

## Highlights

• Multiple organic and metallic particulate matter were observed in BALF
• Particulate matter increases the risk of pulmonary tuberculosis
• Particulate matter affects DNA methylation and cytokine expression
• DNA methylation plays a mediation role between PM and TB

## Graphical abstract

**Figure:**
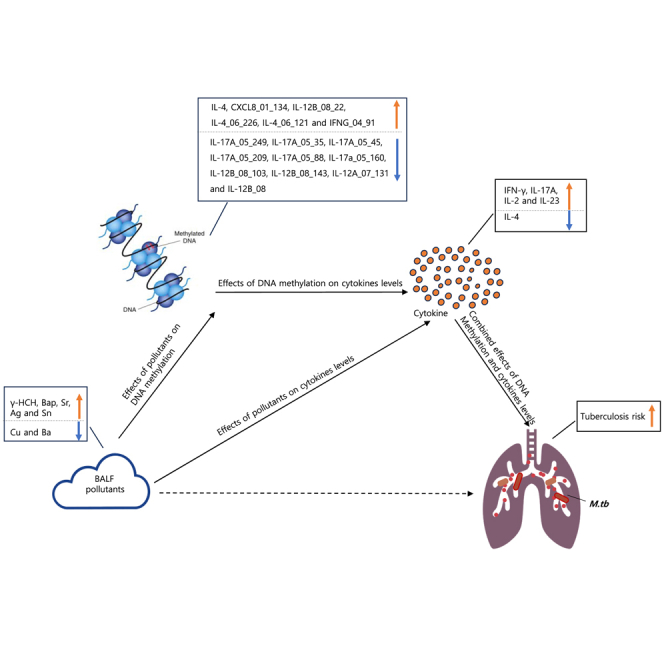

## Introduction

Tuberculosis is an infectious disease caused by *Mycobacterium tuberculosis* (*M.tb*). The causes of active tuberculosis are very complex, including bacterial strains, host immunity, social and environmental factors, and so on.^1^^,^^2^^,^^3^^,^^4^ Many studies have reported the association between air pollution and tuberculosis risk. Exposure to particulate matter (PM), nitrogen dioxide (NO_2_), sulfur dioxide (SO_2_), carbon monoxide (CO), and ozone (O_3_) was associated with an increment in the incidence of newly diagnosed pulmonary tuberculosis (PTB) cases. Long-term exposure to PM_2.5_ would increase the risk of death from tuberculosis and other diseases among tuberculosis patients.^5^^,^^6^^,^^7^^,^^8^

PM is a primary concern of air pollutants and particles with aerodynamic diameter <2.5 μm (PM_2.5_) can penetrate deeply into the terminal bronchioles and proximal alveoli with significant potential for adversely affecting health.^9^^,^^10^^,^^11^ Although several ecological studies have noted a relationship between PM and tuberculosis risk,^12^^,^^13^^,^^14^ the biological mechanism for this association is not fully demonstrated. Direct oxidant generation by PM was attributed to organic and metal components, which might lead to cell injury or apoptosis.^15^ Experimental studies have suggested an impairment of the bacterial clearance process in animals exposed to PM.^16^ However, prior studies investigating the relationship between PM and tuberculosis risk estimated exposure through environmental monitoring sites or personal equipment. This design does not directly evaluate individual-level exposure to PM but reflects the PM concentration of the external environment. Bronchoalveolar lavage (BAL) retrieves secretions that coat the apical surfaces of the bronchial and alveolar epithelium, diluted by saline when performing BAL.^17^ By measuring the organic and metal pollutants in the BALF, evaluating individual exposure to PM can be more accurately measured.

Although the exact mechanisms of PM remain uncertain, recent studies have suggested that epigenetic changes, especially DNA methylation, may help us understand the adverse health effects of PM.^18^^,^^19^ The immune system has multiple mechanisms to remain responsive to environments. Innate and adaptive immune responses take advantage of epigenetic modification to regulate gene expression and maintain long-term phenotypes.^20^ DNA methylation can further affect inflammatory responses, immune alterations, and the risk of diseases.^21^

Currently, most studies are limited to the separate association between air pollutant exposure and DNA methylation, air pollutant exposure, and cytokine concentration. Few utilize mediation analysis to explore the mediation effect of DNA methylation or cytokine concentration in the pollutants affecting tuberculosis risk. To investigate and fill these knowledge gaps, we performed a case-control study to evaluate the associations between air pollutants and PTB risk by detecting organic and metal contaminants in BALF. Further, we investigated the potential mediating effects of DNA methylation and cytokine levels.

## Results

### Demographic characteristics

A total of 132 PTB patients and 30 control patients were enrolled in our study. Of these, 59.8% and 76.7% of PTB patients and controls were male. The mean ages of the PTB and control groups were 55.10 years and 39.65 years, respectively. The BMI of the PTB group was 21.28 kg/m^2^, lower than the control group (22.90 kg/m^2^, p = 0.009). No significant differences were found between PTB and the control group in gender, education level, occupation, diabetes history, fuel type, smoking, and drinking status (Table 1).

**Table 1:** Demographic characteristics of participants at enrollment

Variables	Cases N = 132, n(%)	Controls N = 30, n(%)	χ^2^	*P*
Mean age, years (±SD)	55.10 ± 19.39	39.65 ± 16.53		<0.001
Gender			2.288	0.130
Male	79 (59.8)	23 (76.7)		
Female	53 (40.2)	7 (23.3)		
Body mass index (kg/m^2^)	21.28 ± 2.94	22.90 ± 3.40		0.009
Education level			3.113	0.211
Primary school or lower	16 (12.1)	7 (23.3)		
Middle and high schools	60 (45.5)	14 (46.7)		
College or higher	56 (42.4)	9 (30.0)		
Marital status			2.477	0.116
Unmarried	44 (33.3)	5 (16.7)		
Others	88 (66.7)	25 (83.3)		
Occupation			3.478	0.062
Non-agricultural	107 (81.1)	19 (63.3)		
Agricultural	25 (18.9)	11 (36.7)		
Smoking status			<0.001	0.999
Never	91 (68.9)	21 (70.0)		
Ever	41 (31.1)	9 (30.0)		
Drinking status			0.156	0.692
Never	103 (78.0)	25 (83.3)		
Ever	29 (22.0)	5 (16.7)		
Fuel type			<0.001	0.999
Clean fuel	112 (84.8)	25 (83.3)		
Grubbier fuel	20 (15.2)	5 (16.7)		
Diabetes			0.796	0.372
Yes	12 (9.1)	5 (16.7)		
No	120 (90.9)	25 (83.3)		

### Concentrations of PAHs, OCPs, and metal pollutants in BALF

**Figure 1:**
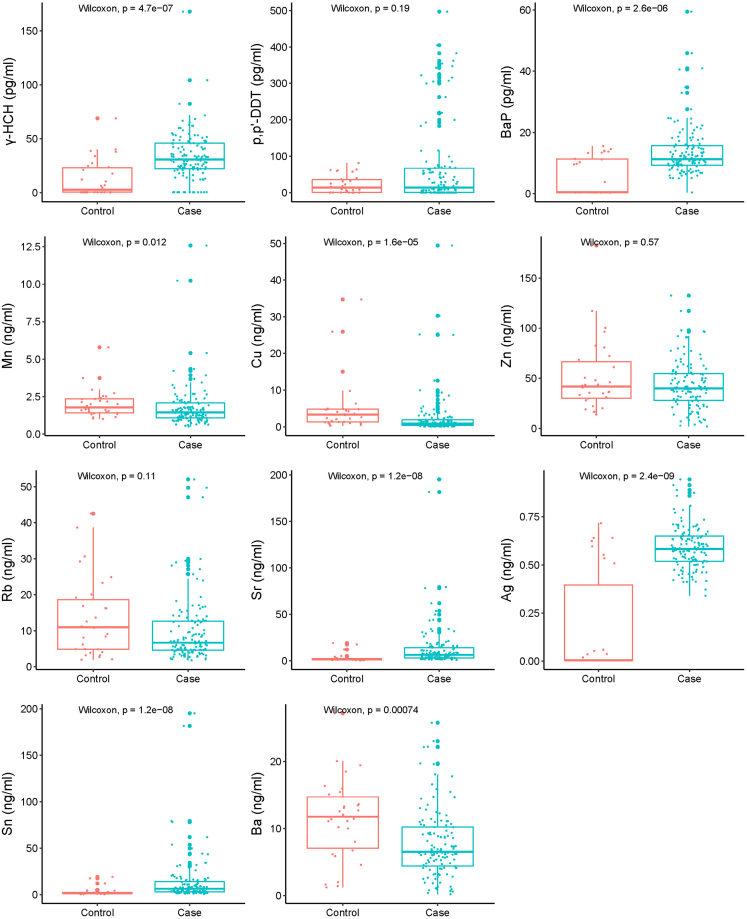
Distribution of air pollutants in bronchoalveolar lavage fluid by different groups

We utilized GC-MS/MS to analyze 25 types of polycyclic aromatic hydrocarbons (PAHs) and OCPs,^22^^,^^23^^,^^24^ and three of them were detected in BALF samples, including gamma-hexachlorocyclohexane (γ-HCH), *p,p*'-DDT, and BaP. Using the established method,^25^ Cr, Mn, Co, Ni, Cu, Zn, As, Rb, Sr, Mo, Ag, Cd, Sn, Ba, and Pb were determined, and among them, Mn, Cu, Zn, Rb, Sr, Ag, Sn, and Ba were detected in BALF samples. The median concentration of γ-HCH in the PTB group was 31.06 pg/mL (IQR: 22.08, 45.81) and 2.53 pg/mL (IQR: 0.36, 22.96) in the control group, with a statistically significant difference between the two groups (p < 0.001). Likewise, the concentration of BaP in the PTB group was higher than in the control group (p < 0.001). The median concentration of Mn was 1.44 ng/mL (IQR: 1.07, 2.03) in the PTB group and 1.77 ng/mL (IQR: 1.41, 2.34) in the control group, with a statistically significant difference (p = 0.010). Similar to Mn, the concentration of Cu and Ba was higher in the control group. Besides, the concentration of Sr, Ag, and Sn was higher in the PTB group (all p < 0.01) (Figure 1; Table S4).

There was statistically increased risk of PTB from γ-HCH (aOR: 1.78; 95% CI: 1.40–2.33), Bap (aOR: 3.46; 95% CI: 2.21–6.45), Sr (aOR: 4.32; 95% CI: 2.33–9.18), Ag (aOR: 8.21; 95% CI: 3.08–81.84), and Sn (aOR: 4.32; 95% CI: 2.33–9.18). There was a protective effect against PTB for Cu (aOR: 0.54; 95% CI: 0.36–0.79) and Ba (aOR: 0.51; 95% CI: 0.25–0.93) (Table 2).

**Table 2:** Multivariable analysis assessing air pollutants associated with pulmonary tuberculosis

Pollutants	aOR	95% CI	*P*
γ-HCH	1.78	1.40–2.33	<0.001
BaP	3.46	2.21–6.45	<0.001
Cu	0.54	0.36–0.79	<0.001
Sr	4.32	2.33–9.18	<0.001
Ag	8.21	3.08–81.84	0.005
Sn	4.32	2.33–9.18	<0.001
Ba	0.51	0.25–0.93	0.042

aOR, adjusted odds ratio, adjusted for age, gender, smoking status, and fuel type; CI, confidence interval.

### Relationship between cytokines level and PTB risk

**Figure 2:**
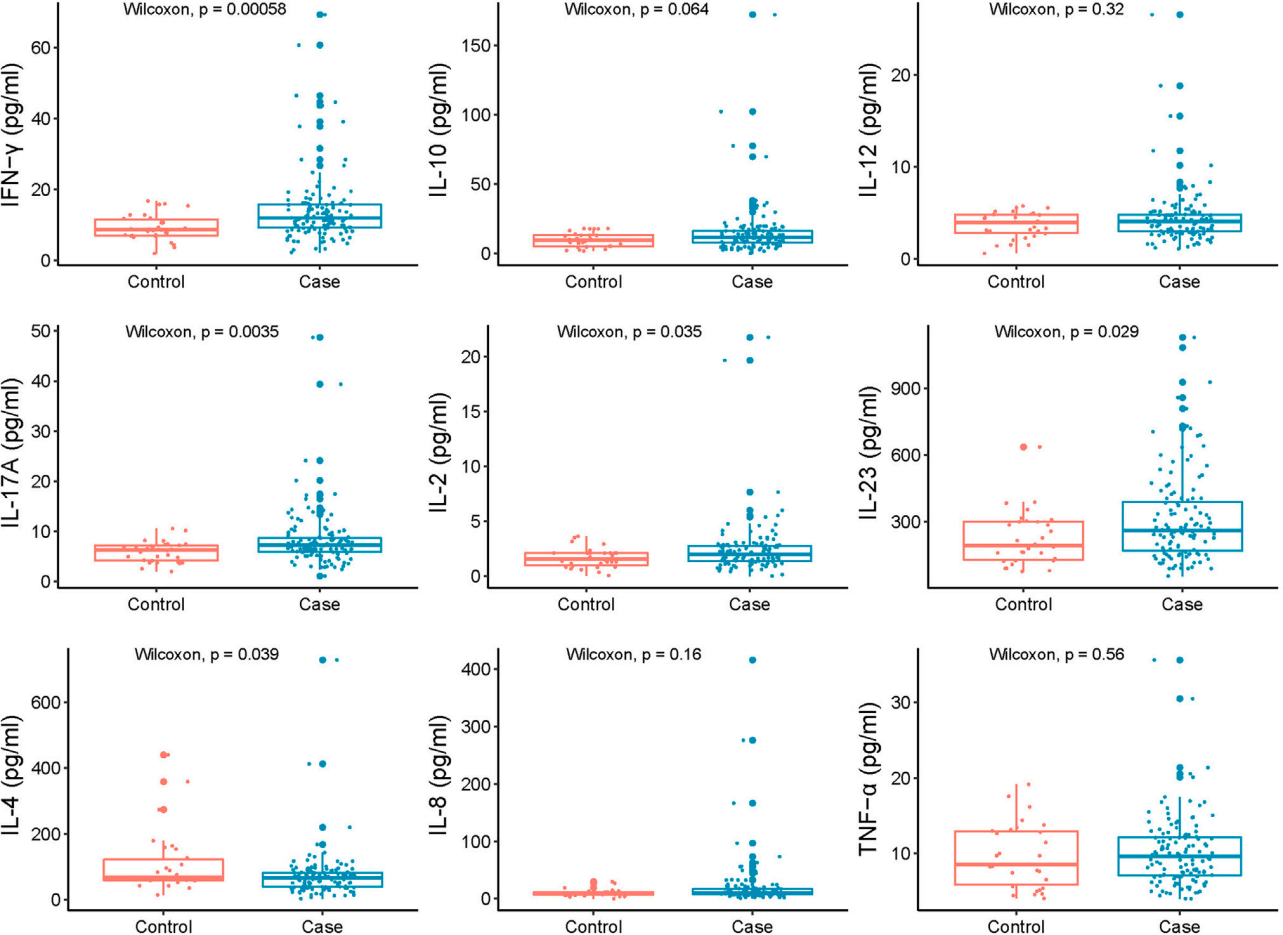
Distribution of cytokines in the plasma of different groups

The concentration of cytokines in plasma was detected through Luminex liquid suspension chip, including IFN-γ, IL-10, IL-12, IL-17A, IL-2, IL-23, IL-4, IL-8, and TNF-α (Figure 2; Table S5). The median concentration of IFN-γ in the PTB group was 12.02 pg/mL, which was higher than that (8.73 pg/mL) in the control group (p < 0.001). The median concentration of IL-17A was higher in the PTB group than in the control group (7.24 versus 6.32 pg/mL; p = 0.004). The median concentration of IL-2 was higher in the PTB group than in the control group (2.01 versus 1.54 pg/mL; p = 0.035). In addition, the concentration of IL-23 was higher in the PTB compared to the control group (260.16 versus 193.65 pg/mL; p = 0.030). Conversely, the concentration of IL-4 was lower in the PTB group than in the control group (65.90 versus 68.21 pg/mL; p = 0.039).

Multivariable logistic regression revealed that IFN-γ (aOR: 2.38; 95% CI: 0.97–5.88), IL-17A (aOR: 2.27; 95% CI: 0.87–5.89), and IL-8 (aOR: 1.67; 95% CI: 0.93–2.99) were suggestively but not statistically related to PTB. IL-4 is protective against PTB, with an aOR of 0.32 (95% CI: 0.12–0.71) (Table S6).

### DNA methylation of immunity-related genes and PTB risk

We sequenced 99 CpG sites in the promoter regions of candidate genes. Methylation levels of target genes among PTB and control groups can be seen in Figure S2. The methylation levels of IFNG, IL-12B, IL-17A, and IL-4 (accounting for 57% of the total number of detected genes) were statistically distinct between the two groups (p < 0.05). There was no significant difference in CXCL8, IL-12A, and IL-23A methylation levels. Among the 99 CpG sites, 26 of them were statistically significant between the two groups (p < 0.05), accounting for 26.3% of the total number of detected CpG sites (Table S7).

Methylation levels of IL-4, CXCL8_01_134, IL-12B_08_22, IL-4_06_226, IL-4_06_121, and IFNG_04_91 were positively correlated with the risk of PTB (all p < 0.05) (Table 3). The methylation level of IL-17A_05_249, IL-17A_05_35, IL-17A_05_45, IL-17A_05_209, IL-17A_05_88, IL-17A_05_160, IL-12B_08_103, IL-12B_08_143, IL-12A_07_131, and IL-12B_08 was negatively correlated with the risk of PTB (p < 0.05).

**Table 3:** Multivariable logistic regression analysis to investigate DNA methylation levels and pulmonary tuberculosis

CpG sites	aOR	95% CI	*P*
CXCL8_01_134	1.67	1.06–2.72	0.030
IFNG_04_91	1.11	1.01–1.23	0.038
IL-12A_07_131	0.17	0.04–0.70	0.018
IL-12B_08_22	2.66	1.27–6.19	0.015
IL-12B_08_103	0.43	0.20–0.90	0.025
IL-12B_08_143	0.29	0.12–0.66	0.004
IL-17A_05_35	0.71	0.51–0.96	0.034
IL-17A_05_45	0.71	0.53–0.92	0.015
IL-17A_05_88	0.62	0.41–0.89	0.015
IL-17A_05_160	0.62	0.46–0.81	0.001
IL-17A_05_209	0.70	0.55–0.86	0.001
IL-17A_05_249	0.79	0.67–0.91	0.002
IL-4_06_121	1.25	1.12–1.41	<0.001
IL-4_06_226	1.34	1.09–1.68	0.007
IFNG_04 gene fragment	1.12	1.02–1.25	0.026
IL-12B_08 gene fragment	0.06	0.01–0.68	0.029
IL-4_06 gene fragment	2.15	1.38–3.58	0.002
IL-4 gene	1.93	1.28–3.06	0.003

aOR, adjusted odds ratio, adjusted for age, gender, smoking status, and fuel type; CI, confidence interval.

### Assessing DNA methylation as a mediator to the association between air pollutant exposure and PTB risk

The effect of air pollutant exposure on DNA methylation was analyzed using a linear regression model, considering age, gender, smoking status, and fuel type as covariates. The methylation level of CXCL8_01_43 and CXCL8_01_88 sites was correlated with Sn, Sr, Mn, and γ-HCH. Table S8 also showed the association between CpG sites of IFNG, IL-12A, IL-12B, IL-17A, and IL-4 and pollutants with statistical significance (p < 0.05).

Likewise, we included age, gender, smoking status, and fuel type as covariates. In the association between Sr, Sn, γ-HCH, and PTB risk, the mediation effect of IL-4_06_121 level was significant, with the estimated ($a_{1}\times b_{1}$) of 0.16 (95% CI: 0.01–0.56), 0.16 (95% CI: 0.01–0.56), and 0.11 (95% CI: 0.01–0.44), respectively. When converting to aORs, the mediating effect values were 1.22 (95% CI: 1.01–1.76), 1.22 (95% CI: 1.01–1.76), and 1.15 (95% CI: 1.01–1.55), respectively (Table 4).

**Table 4:** Mediation effect of DNA methylation in the association between pollutant exposure and pulmonary tuberculosis risk

Pollutant	CpG sites	Mediation effect (95% CI)	aOR (95% CI)
Sr	IL-4_06_121	0.16(0.01–0.56)	1.22(1.01–1.76)
Sn	IL-4_06_121	0.16(0.01–0.56)	1.22(1.01–1.76)
γ-HCH	IL-4_06_121	0.11(0.01–0.44)	1.15(1.01–1.55)

aOR, adjusted odds ratio, adjusted for age, gender, smoking status, and fuel type; CI, confidence interval.

### Assessing cytokines levels as a mediator to the association between DNA methylation and PTB risk

We hypothesized that DNA methylation may impact PTB risk through cytokine regulation. As shown in Table S9, IL-4 significantly mediated the association between three CpG sites of the IL-4 gene and PTB risk, with age, gender, smoking status, and fuel type as covariates. The estimates of the mediation effect ($a_{2}\times b_{2}$) were smaller than 1.000, suggesting that increased methylation levels of the IL-4 gene and decreased IL-4 concentrations might increase the risk of PTB.

### Assessing cytokines levels as a mediator to the association between air pollutant exposure and PTB risk

The effect of the pollutant on cytokines level was analyzed by the linear regression model, considering age, gender, smoking status, and fuel type as covariates. The concentration of γ-HCH, BaP, and Ag was positively correlated with IL-17A, and the estimates of coefficient $a_{3}$ were 0.49 (95% CI: 0.02–0.95), 0.79 (95% CI: 0.05–1.54), and 0.70 (95% CI: 0.11–1.29), respectively. γ-HCH, BaP, *p,p*’-DDT, Sr, Ag, and Sn concentrations were negatively correlated with IL-4 levels (all p < 0.05).

However, our hypothesis that cytokine levels may mediate the relationship between air pollutants and PTB risk was not substantiated in this analysis.

## Discussion

In this study, we measured individual-level concentration exposures of organic pollutants and metals in BALF from patients suspected of PTB. We found significant differences in the distribution of pollutants between PTB cases and controls for the first time. We also identified a complex relationship between air pollutant exposure, methylation status of immune-related genes, cytokine levels, and PTB risk.

Our study first determined the concentration of PAHs, OCPs, and metal pollutants in BALF of the PTB patients. Almost all studies on PM_2.5_ estimate individual PM_2.5_ exposure through air sampling, environmental monitoring points, or personal monitors, which cannot truly assess the exposure level of PM_2.5_ in the body. Specifically, by measuring the pollutants in the BALF, the exposure level of PM_2.5_ can be reflected. The results showed that the concentration of γ-HCH, *p,p*'-DDT, and BaP in the PTB group was higher than in the control group. After adjusting for factors (age, gender, smoking status, and fuel type), γ-HCH and Bap remained risk factors for PTB. As a widely used and persistent insecticide, γ-HCH was highly toxic and non-degradable.^26^ Previous studies on γ-HCH residues mainly focused on soil, air, and water samples. Fang et al.^27^ conducted a survey in China to evaluate OCPs residues in soil and vegetation, showing that HCH was the dominant OCPs detected. It was also found that the activity of superoxide dismutase increased with the dose of *p,p*'- DDT and γ-HCH in mice experiments,^28^ and the release of the IL-6 increased after *p,p*'- DDT stimulated the immune cells.^29^ In addition to being a potent chemical carcinogen, BaP is also an immunosuppressant in mammals.^30^^,^^31^ Uno et al.^32^ found that BaP can be metabolized into active compounds to form DNA adducts and induce cells to produce reactive oxygen species, further leading to the production of inflammatory cytokines.

In addition to organic pollutants, eight metal pollutants were also detected in BALF, among which the concentration of Sr, Ag, and Sn were higher in PTB than in controls and were risk factors for PTB. Heavy metals carried by PM can induce oxygen free radicals and stimulate oxidative stress reactions. Similarly, transition metals can also provide electrons to form superoxide and hydrogen peroxide and directly consume endogenous mercaptan antioxidants.^15^^,^^33^^,^^34^ Studies have found that metal elements such as Cd, Hg, Pb, Ni, and Zn in PM_2.5_ could inhibit the cellular immune function of mice, manifested by changes in lymphocyte transformation, interleukin activity, natural killer cell activity, and T lymphocyte subsets.^35^^,^^36^^,^^37^ In this study, we detected some organic and inorganic pollutants in BALF, which are part of the PM_2.5_ component, so we did not directly compare the results with airborne PM_2.5_.

We also found that the IFN-γ, IL-17A, and IL-23 levels in the plasma of PTB were statistically higher than those in controls. Exposure to γ-HCH, BaP, and Ag *in vivo* was positively correlated with IL-17A. The concentration of γ-HCH, BaP, *p,p*’-DDT, Sr, Ag, and Sn were negatively correlated with IL-4 level, and the lower the IL-4 level, the higher the PTB risk. IL-4 is secreted by Th2 cells and can inhibit the production of other cytokines, including IL-1, IL-6, IL-8, and TNF-α, as well as the production and migration of lymphocytes and macrophages. Previous studies showed that the level of IL-4 in tuberculosis patients was decreased,^38^^,^^39^ suggesting that IL-4 may be associated with the development of tuberculosis. Gao et al.^40^ found that short-term exposure to air pollutants increased systemic inflammatory response, which may be related to the release of pro-inflammatory cytokines, including IL-2, IL-12 IL-17A, and IFN-γ, and decreased anti-inflammatory cytokines levels, including IL-4 and IL-13. Above all, exposure to air pollutants might activate inflammatory pathways and change the ratio of pro-inflammatory factors to anti-inflammatory factors, which may aggravate the high-intensity inflammatory response in the lungs and increase the risk of PTB.

DNA methylation played a role in regulating the expression of immune-related genes, and hypermethylation of promoter region was considered to be one of the potential molecular mechanisms of gene silencing.^41^ Lee et al.^42^ found that the IL-4 gene underwent a series of complex methylation and demethylation steps during helper T cell differentiation. However, Falek et al.^43^ found that the IFN-γ and IL-4 were not related to methylation levels of these two genes. At present, there are still inconsistent conclusions between DNA methylation and IL-4 expression, which needs to be further studied. Exposure to air pollutants could affect DNA methylation levels and further influence inflammatory responses, immune changes, and disease risk.^21^ The air pollution components related to traffic, such as PM_2.5_, O_3_, NO_X_, and PAHs, are associated with DNA methylation.^21^ A study showed that exposure to PM_2.5_ was correlated with the methylation of CpG sites of IL-4, IL-10, and IFN-γ.^44^ In this study, except for IL-4_06_150, other CpG sites level of IL-4 increased with the increase in γ-HCH, Bap, Sn, Sr, Ba, Rb, and Ag concentration, which may be related to the exposure time and intensity of the pollutant.

Mediation analysis showed that the methylation level of IL-4_06_121 mediated the association between Sr, Sn, and γ-HCH and PTB risk, and IL-4 was a significant mediator between the methylation level of the IL-4 gene and PTB risk. We concluded that exposure to air pollutants in BALF may affect the risk of PTB by the mediation effect of the methylation level IL-4 gene and IL-4 gene expression. Wang et al.^45^ used the mediation model to analyze the role of DNA methylation between short-term PM_2.5_ exposure and damage to inflammatory markers. Janssen et al.^46^ found that 27% of the effect of changes in placental mitochondrial DNA content caused by PM_2.5_ exposure during pregnancy was mediated by D loop gene methylation. Another study found that 22.13% of the effects of PAH exposure on lung function decline were mediated by low levels of CC16 in plasma.^47^

### Conclusions

In conclusion, by measuring individual-level concentration exposures of organic pollutants and metals in BALF, we found significant differences in the distribution of pollutants between PTB cases and controls. We found a complex relationship between air pollutant exposure, methylation status of immune-related genes, cytokine levels, and the risk of PTB.

### Limitations of the study

This study is the first to detect air pollutant exposure in BALF of PTB patients. Further, it analyzes the association between pollutants and PTB and the effects of air pollutants on immunity. However, the shortcomings of this study should not be ignored. First, BAL is an invasive examination; only patients with suspected lung disease were selected as controls. Due to this, our control groups were not healthy. Although there is no apparent lung damage in patients with mild pneumonia or bronchitis, there is still inflammation in the lung, which may cause changes in the retention of pollutants and cytokines in the blood. Thus, the concentration of pollutants in the control group may be somewhat underestimated, and misleading information was unavoidable. Furthermore, the size of the control group was small. A larger sample size in the control group will improve the statistical efficiency of our study, making the results more reliable. Therefore, our results need to be confirmed in future research. Second, detected pollutants, cytokines, and DNA methylation were limited, potentially impacting their relationships. Third, other epigenetic changes may also affect gene expression and DNA methylation. Further work is needed to understand the role of epigenetic changes other than DNA methylation concerning PTB risk.

## STAR★Methods

### Key resources table

**Table:** 

REAGENT or RESOURCE	SOURCE	IDENTIFIER
**Biological samples**		

BALF and blood samples	Nanjing Public Health Medical Center	/

**Chemicals, peptides, and recombinant proteins**		

Organochlorine pesticide mix	Merck Sigma-Aldrich	https://www.sigmaaldrich.com/SG
CLMS-2 mix	Spex CertiPrep	https://www.spex.com/Product

**Others**		

Gas chromatography-mass spectroscopy	Thermo Scientific Co., USA	https://assets.thermofisher.com
Agilent 7900 ICP-MS	Agilent Technologies, Santa Clara, USA	https://www.agilent.com
Elemental Inorganic Standards	Agilent Technologies, Santa Clara, USA	https://www.agilent.com
The Human High Sensitivity T cell Magnetic Bead Panel	Millipore Corporation, Billerica, MA, USA	https://www.merckmillipore.com
Luminex 200 system	Luminex Corporation, Austin, TX, USA	https://www.luminexcorp.com/luminex-100200

### Resource availability

#### Lead contact

Further information and requests for resources and reagents should be directed to and will be fulfilled by the lead contact, Qiao Liu (liuqiaonjmu@163.com).

#### Materials availability

This study did not generate new unique reagents.

#### Data and code availability

• Data reported in this paper will be shared by the lead contact upon request.
• This paper does not report the original code
• Any additional information required to reanalyze the data reported in this paper is available from the lead contact upon request

### Experimental model and study participant details

Our study does not use experimental models. We enrolled 132 patients and 30 controls who received BALF at Nanjing Public Health Medical Center from September 2018 to June 2019. According to bacteriological examinations (sputum/BALF smear, sputum/BALF culture, and sputum/BALF GeneXpert MTB/RIF), radiological results, and clinical characteristics, subjects were divided into PTB group and control group. PTB was diagnosed according to China’s National Diagnostic Criteria for PTB (WS 288–2017). The inclusion criteria for PTB cases were: living in Nanjing for the past year; sputum samples are available for sputum smear and culture; no antituberculosis therapy at enrollment; HIV negative; willingness to participate in this program. The inclusion criteria of the control group were: living in Nanjing for the past year; sputum samples are available for sputum smear and culture; no current or prior PTB; HIV negative; willingness to participate in this program. Trained epidemiologists administered each participant a baseline interview. The questionnaire included demographic indicators, body mass index (BMI), education level, tobacco smoking, alcohol drinking, household fuel, and history of tuberculosis. Blood and BALF samples were collected, stored, and refrigerated at −80°C for further testing.

### Method details

#### Air pollutant exposure assessment

BALF samples were collected to estimate *in vivo* organic and metal pollutants exposure. Since the dilution used in BALF was 0.9% normal saline, comparing the differences between the concentration of Na in each sample and in normal saline can determine the dilution multiple of target pollutants. By reading the absorbance of each sample and comparing it with the standard curve, the concentration of Na was further analyzed through an atomic absorption spectrometer (contrAA700, Jena, German). The concentration of organochlorine pesticides (OCPs) and PAHs was detected by gas chromatography-mass spectroscopy (GC-MS/MS, TSQ80000, Thermo Scientific Co., USA). We used dichloromethane as the extraction medium to extract the constituents from the BALF samples. Deuterated PAH standard mixtures, including naphthalene-d_8_ (Nap-d_8_), acenaphthene-d_10_ (Acp-d_10_), phenanthrene-d_10_ (Phe-d_10_), and Benz (a) anthracene-d_12_ (BaA-d_12_) were served as recovery surrogate standards (Sigma–Aldrich, USA). A total of 16 PAHs and 10 OCPs were tested using an internal standard and quantified roughly by peak area. The retention times of individual standards of PAHs and OCPs are described in Table S1.

The instrument Agilent 7900 ICP-MS (Agilent Technologies, Santa Clara, USA) was utilized for the metal elemental analysis of the BALF. The ICP-MS instrument was calibrated using standards of elements (Agilent Technologies, Santa Clara, USA). The internal standard solution (including yttrium, rhodium, rhenium, Spex, USA) was diluted with 1% nitric acid to 100 μg/L. Besides, all samples were stabilized and diluted in 1% nitric acid. The instrumental setting details for the analysis are given in Table S2. The recovery rate was 85%–105%, with a relative standard deviation of less than 10%.

#### Luminex liquid suspension chip detection

Luminex liquid suspension chip detection was performed by Wayen Biotechnologies (Shanghai, China). The Human High Sensitivity T cell Magnetic Bead Panel (Millipore Corporation, Billerica, MA, USA) was utilized in accordance with the manufacturer’s instructions. First, 50 μL background, standard, and quality controls were added to their appropriate wells. Second, a 25 μL plasma sample and 25 μL assay buffer were added to the sample wells, resulting in a 2-fold sample dilution. After the bead captured an analyte from a test sample, a biotinylated detection antibody was introduced on the assay plate. The reaction mixture was then incubated with Streptavidin-PE conjugate, the reporter molecule, to complete the reaction on the surface of each microsphere. Subsequently, values were read using the Luminex 200 system (Luminex Corporation, Austin, TX, USA).

#### DNA extraction and gene-specific DNA methylation measurement

Genomic DNA was extracted from peripheral blood samples using the Tiangen DNA kit (Tiangen Biotech, Beijing, China) according to the manufacturer’s instructions. We selected seven PTB immunity-related genes (CXCL8, IFNG, IL-12A, IL-12B, IL-17A, IL-23A, IL-4)^48^^,^^49^^,^^50^ and sequenced the CpG islands in the promoter region of the candidate genes through the Illumina MiSeq platform. Briefly, primers were designed to amplify the regions of interest from the bisulfite-converted DNA (Table S3), and DNA samples were bisulfite-treated by employing the EZ DNA Methylation-GOLD Kit (Zymo Research, Orange, CA, USA). A 20 μL PCR reaction mixture was prepared for multiplex PCR with the cycling program of 95°C for 2 min; 11 cycles of 95°C for 20 s, 62°C for 30 s with a decreasing temperature step of 0.5°C per cycle, and 72°C for 0.5 min; 1 cycle of 72°C for 3 min; and 4°C forever. All PCR products were quantified, pooled, and then subjected to the Illumina MiSeq platform with 2 × 150 bp double-terminal sequencing mode.

#### Ethic approves

This study was approved by the Ethics Committee of Nanjing Medical University. After informed consent was obtained from all participants, questionnaires were used to collect demographic data.

### Quantitation and statistical analysis

We summarized continuous variables as medians with interquartile ranges (IQRs) and categorical variables as frequencies (n) and proportions (%). Pearson χ^2^ or Fisher’s exact tests were used as appropriate to derive meaningful differences between categorical variables. Comparisons between groups were performed using *t* or ANOVA tests if continuous variables were normally distributed. Otherwise, non-parametric tests for group comparisons. We used binary logistic regression models to evaluate associations between pollutants and PTB risk. The odds ratios and their 95% confidence intervals (CIs) were used to estimate the strength of the association. The linear regression model was utilized if the outcome variable was continuous. All statistical tests were two-tailed, and the significance level was set at 0.05.

We conducted further analysis considering that DNA methylation and cytokine level may mediate the associations between pollutant exposure and PTB risk. We established three distinct mediation models to account for the possibility of mediation (Figure S1).^51^ Two basic models were applied, and their estimates were used as inputs for the mediating function. The first model assessed the effects of pollutants on DNA methylation ($a_{1}$), including age, gender, smoking status, and fuel type as covariates. The second model assessed the combined effects of pollutants and the mediator (DNA methylation) on PTB, where $b_{1}$ was the coefficient of the mediator on the outcome. Two basic models were corrected using the False Discovery Rate.^52^ A total of 1,000 bootstraps were run to estimate the 95% CI of the mediation effect ($a_{1}\times b_{1}$). We conducted the same steps to assess cytokine levels as a mediating factor for the relationship between (i) DNA methylation and PTB ($a_{2}\times b_{2}$) and (ii) pollutants and PTB ($a_{3}\times b_{3}$).

### Additional resources

Any additional information about the simulation and data reported in this paper is available from the lead contact on request.
