# Supplementary material for: Air pollutants in bronchoalveolar lavage fluid and pulmonary tuberculosis: A mediation analysis of gene-specific methylation
Source: iScience. 2023 Nov 3;26(12):108391. doi: 10.1016/j.isci.2023.108391 (PMC10690542; doi:10.1016/j.isci.2023.108391)
Supplement: Document S1. Figures S1, S — 2, and Tables S1–S9 [file mmc1.pdf]

iScience, Volume *26*

Supplemental information

Air pollutants in bronchoalveolar lavage fluid and pulmonary tuberculosis: A mediation analysis of gene-specific methylation

Qiao Liu, Ye Ji, Li Wang, Zhongqi Li, Bilin Tao, Limei Zhu, Wei Lu, Leonardo Martinez, Yi Zeng, and Jianming Wang

Supplementary files

Table S1. Retention time, ion pairs, and of the target substance and isotope internal standard, related to Figure 1and Table 2.

Pollutants	Retention time (min)	Parent ion (m/z)	Daughter ion (m/z)	Collision energy (eV)	Isotope internal standard
Nap	10.33	128	78	20	Nap-d_8
		128	102	16	
Ace	13.47	152	126	20	
		152	151	18	
Acp	13.78	154	152	26	Acp-d_{10}
		154	153	10	
FI	14.68	165	139	24	
		165	164	12	
a-HCH	15.56	181	145	16	
		183	147	14	
b-HCH	16.00	181	145	14	
		183	147	14	
c-HCH	16.07	183	147	14	
		181	145	14	
Phe	16.33	178	151	26	
		178	176	20	Phe-d_{10}
An	16.42	178	151	26	
		178	176	20	
d-HCH	16.53	181	145	14	
		183	147	14	
Flu	18.29	202	176	32	
		202	200	30	
o,p'-DDE	18.36	246	176	34	
		318	248	20	
		202	200	26	

Pyr	18.64	202	201	16	
p,p'-DDE	18.74	246	176	30	
		318	248	20	
o,p'-DDD	18.83	199	163	30	
		235	165	22	
p,p'-DDD	19.22	235	165	19	
		235	199	13	
o,p'-DDT	19.24	235	165	22	
		235	199	13	
p,p'-DDT	19.60	235	165	22	
		235	199	15	
BaA	20.21	228	224	38	BaA-d_{12}
		228	226	22	
Chr	20.26	228	202	25	
		228	226	26	
BbF	21.44	252	226	24	
		252	250	26	
BkF	21.47	252	226	26	
		252	250	32	
BaP	21.85	252	226	25	BaA-d_{12}
		252	250	30	
DBA+BghiP	23.41	276	250	35	
		276	274	30	
InP	23.88	276	272	25	
		276	274	26	

Table S2. Instrument Parameters for the ICP-MS-based elemental analysis of bronchoalveolar lavage fluid, related to Figure 1 and Table 2.

Instrument parameters	Values
Radio-frequency power (W)	1550
Plasma gas flow rate (L/min)	15.0
Atomizing gas flow rate (L/min)	0.99
Scan times (time)	60
Dwell time (ms)	100
Repeated measurement (time)	3

Table S3. Primers designed for sequenced CpG sites of immunity-related selected genes, related to Table 3 and Table 4.

Gene fragment	Gene	Length (bp)	Forward primer	Reverse primer
CXCL8_01	CXCL8	186	GGTGGTTTAAGTTTGTAATTTTAGTATTTTG	CATTCTCCTACCTCAACCTCCT
IFNG_03	IFNG	147	AATTAGTYGGGYKTGGYGAGTGTT	AAACRAAATCTTACTCTATCACCCAAAC
IFNG_04	IFNG	144	AAAGAAATGATTAGGTYGGGTG	TTATACTATTTTTAATACAAACRAAATTTCA
IL-12A_07	IL-12A	227	GGGTGTTTYGTTYGTTTGTGGA	AACTCTAATCTCTTACTTTCTATCTCCTTCT
IL-12B_08	IL-12B	213	AGGGATGGAGAAGTGGATTTG	TCCCCCRCCCAATCTACAC
IL-17A_05	IL-17A	276	TTTAATTAAAATGGTGTTATTTTTGAAT	AATCAAAACCCAACRTTTCATACC
IL-23A_02	IL-23A	237	GGYGATTTTAGGAGAGAGYGTGTG	CCAACAACRACTCCTAATACTTTACCT
IL-4_06	IL-4	283	ATTTATAGGGAGGTTTAGGTATAGTGGTT	TTACTCTATCACCCAAACTAAAATACAATA

Table S4. Comparisons of air pollutants in BALF samples between groups, related to Figure 1and Table 2.

**Target substance**	**Detection rate (%)**	**PTB group (n=132), median [IQR]**	**Control group (n=30), median [IQR]**	***P***
γ-HCH, pg/ml	99.24	31.06 [22.08, 45.81]	2.53 [0.36, 22.96]	<0.001
p,p'-DDT, pg/ml	89.39	16.98 [0.40, 69.31]	13.84 [0.39, 35.68]	0.136
BaP, pg/ml	64.39	11.24 [9.34, 15.81]	0.40 [0.36, 11.30]	<0.001
Mn, ng/ml	100.00	1.44 [1.07, 2.03]	1.77 [1.41, 2.34]	0.010
Cu, ng/ml	90.13	0.87 [0.41, 1.95]	3.31 [1.31, 4.78]	<0.001
Zn, ng/ml	100.00	40.50 [28.75, 54.58]	41.69 [30.13, 66.57]	0.605
Rb, ng/ml	100.00	6.64 [4.55, 12.56]	10.96 [4.86, 18.65]	0.100
Sr, ng/ml	100.00	6.43 [2.89, 12.70]	1.51 [1.19, 2.41]	<0.001
Ag, ng/ml	88.81	0.59 [0.52, 0.65]	0.00 [0.00, 0.40]	<0.001
Sn, ng/ml	100.00	6.43 [2.89, 12.70]	1.51 [1.19, 2.41]	<0.001
Ba, ng/ml	100.00	6.76 [4.69, 10.31]	11.78 [7.08, 14.73]	0.001

IQR: interquartile range

Table S5. Comparisons of cytokines in plasma (pg/ml) between groups, related to Figure 2.

**Cytokines**	**PTB group (n=132), median (IQR)**	**Control group (n=30), median (IQR)**	**T**	***P***
IFN-γ	12.02 (9.18, 15.76)	8.73 (7.01, 11.88)	3.443	<0.001
IL-10	11.66 (7.32, 16.22)	9.35 (4.82, 13.38)	1.852	0.064
IL-12	4.07 (2.97, 4.79)	3.92 (2.65, 4.81)	0.990	0.322
IL-17A	7.24 (5.94, 8.83)	6.32 (4.12, 7.29)	2.918	0.004
IL-2	2.01 (1.37, 2.78)	1.54 (0.91, 2.11)	2.114	0.035
IL-23	260.16 (165.02, 394.10)	193.65 (125.76, 300.96)	2.176	0.030
IL-4	65.90 (38.72, 82.56)	68.21 (58.20, 133.61)	-2.061	0.039
IL-8	10.08 (7.53, 17.00)	9.40 (6.52, 11.87)	1.414	0.157
TNF-α	9.60 (7.11, 12.18)	8.54 (5.56, 13.03)	0.586	0.558

IQR: interquartile range

Table S6. Multivariable logistic regression analysis to access cytokines level and pulmonary tuberculosis risk, related to Figure 2 and Table 4.

**Cytokines**	**aOR**	**95% CI**	***P***
IL-4	0.32	0.12-0.71	0.009
IFN-γ	2.38	0.97-5.88	0.060
IL-17A	2.27	0.87-5.89	0.093
IL-8	1.67	0.93-2.99	0.086

aOR: adjusted odds ratio, adjusted for age, gender, smoking status, and fuel type.

Table S7. Methylation levels of CpG sites between groups, related to Table 3 and Table 4.

**CpG sites**	**PTB group, mean±SD**	**Control group, mean±SD**	***t***	***P***
CXCL8_01_134	0.031±0.009	0.026±0.012	2.586	0.011
IFNG_03_65	0.190±0.008	0.184±0.015	3.088	0.038
IFNG_04_33	0.566±0.045	0.550±0.039	2.123	0.027
IFNG_04_66	0.511±0.053	0.486±0.056	2.289	0.023
IFNG_04_70	0.502±0.057	0.479±0.053	2.023	0.045
IFNG_04_77	0.548±0.062	0.512±0.061	2.828	0.005
IFNG_04_91	0.621±0.049	0.588±0.050	3.380	0.001
IL-12A_07_78	0.007±0.004	0.005±0.004	2.493	0.014
IL-12B_08_22	0.015±0.006	0.011±0.008	2.068	0.046
IL-12B_08_143	0.009±0.004	0.013±0.008	-2.551	0.016
IL-17A_05_35	0.950±0.044	0.963±0.013	-2.345	0.021
IL-17A_05_45	0.945±0.019	0.958±0.019	-3.326	0.001
IL-17A_05_88	0.967±0.013	0.974±0.014	-2.858	0.005
IL-17A_05_160	0.933±0.020	0.949±0.018	--3.977	<0.001
IL-17A_05_209	0.929±0.026	0.953±0027	-4.529	<0.001
IL-17A_05_249	0.868±0.036	0.901±0.038	-4.397	<0.001
IL-23A_02_26	0.009±0.005	0.006±0.005	2.309	0.022
IL-23A_02_55	0.007±0.004	0.005±0.005	2.465	0.015

IL-23A_02_142	0.005±0.003	0.004±0.005	2.024	0.046
IL-4_06_32	0.388±0.020	0.378±0.024	2.572	0.003
IL-4_06_60	0.321±0.018	0.315±0.023	2.453	0.023
IL-4_06_76	0.132±0.020	0.121±0.023	2.672	0.008
IL-4_06_110	0.109±0.017	0.101±0.019	2.216	0.028
IL-4_06_121	0.411±0.044	0.347±0.072	6.296	<0.001
IL-4_06_150	0.173±0.018	0.177±0.019	-2.563	0.013
IL-4_06_226	0.230±0.022	0.214±0.025	3.505	0.044

SD: standard deviation

Table S8. Association between pollutants exposure and methylation levels of CpG sites, related to Table 3 and Table 4.

DNA methylation	Pollutants	Beta	SE	Lower 95%	Upper 95%	*P*
CXCL8_01 gene fragment	Mn	-0.47	0.235	-0.931	-0.009	0.047
IFNG_04 gene fragment	Ag	0.743	0.267	0.22	1.266	0.006
IFNG_04 gene fragment	γ-HCH	0.556	0.212	0.14	0.972	0.01
IFNG gene	Ag	0.396	0.151	0.10	0.692	0.009
IFNG gene	Zn	0.631	0.311	-0.551	-0.123	0.044
IFNG gene	γ-HCH	0.281	0.12	0.046	0.516	0.02
IL-12B_08 gene fragment	Ag	-0.025	0.01	-0.045	-0.005	0.012
IL-12B gene	p,p'-DDT	0.036	0.016	0.005	0.067	0.027
IL-12A_07 gene fragment	p,p'-DDT	0.007	0.003	0.001	0.013	0.018
IL-12B_08	BaP	-0.035	0.012	-0.059	-0.011	0.005
IL-17A_05 gene fragment	Ag	-0.477	0.215	-0.898	-0.056	0.028
IL-17A_05 gene fragment	Cu	0.49	0.245	0.01	0.97	0.047
IL-17A gene	Ag	-0.471	0.214	-0.89	-0.052	0.03
IL-17A gene	Cu	0.505	0.244	0.027	0.983	0.04
IL-23A_02 gene fragment	BaP	0.026	0.01	0.006	0.046	0.011
IL-23A_02 gene fragment	Cu	-0.02	0.009	-0.038	-0.002	0.032
IL-23A gene	Ag	0.04	0.011	0.018	0.062	<0.001
IL-23A gene	BaP	0.047	0.014	0.02	0.074	0.001
IL-23A gene	γ-HCH	0.026	0.009	0.008	0.044	0.004
IL-4_06 gene fragment	Ag	0.346	0.06	0.228	0.464	<0.001
IL-4_06 gene fragment	BaP	0.321	0.08	0.164	0.478	<0.001
IL-4_06 gene fragment	Sn	0.221	0.081	0.327	1.401	0.007
IL-4_06 gene fragment	Sr	0.221	0.081	-0.18	-0.034	0.007
IL-4_06 gene fragment	γ-HCH	0.104	0.052	0.002	0.206	0.046
IL-4 gene	Ag	0.358	0.065	0.231	0.485	<0.001
IL-4 gene	BaP	0.343	0.085	0.176	0.51	<0.001
IL-4 gene	Sn	0.228	0.087	0.009	0.083	0.009
IL-4 gene	Sr	0.228	0.087	-0.146	-0.036	0.009
IL-4 gene	γ-HCH	0.115	0.055	0.007	0.223	0.039

Table S9. Mediation effect of cytokines level in the association between DNA methylation and pulmonary tuberculosis risk, related to Table 4.

**CpG sites**	**Cytokines**	**Mediation effect**	**95% CI**
IL-4 gene	IL-4	0.15	0.01-0.35
IL-4_06 gene fragment	IL-4	0.14	0.01-0.35
IL-4_06_121	IL-4	0.09	0.02-0.29

CI: confidence interval.

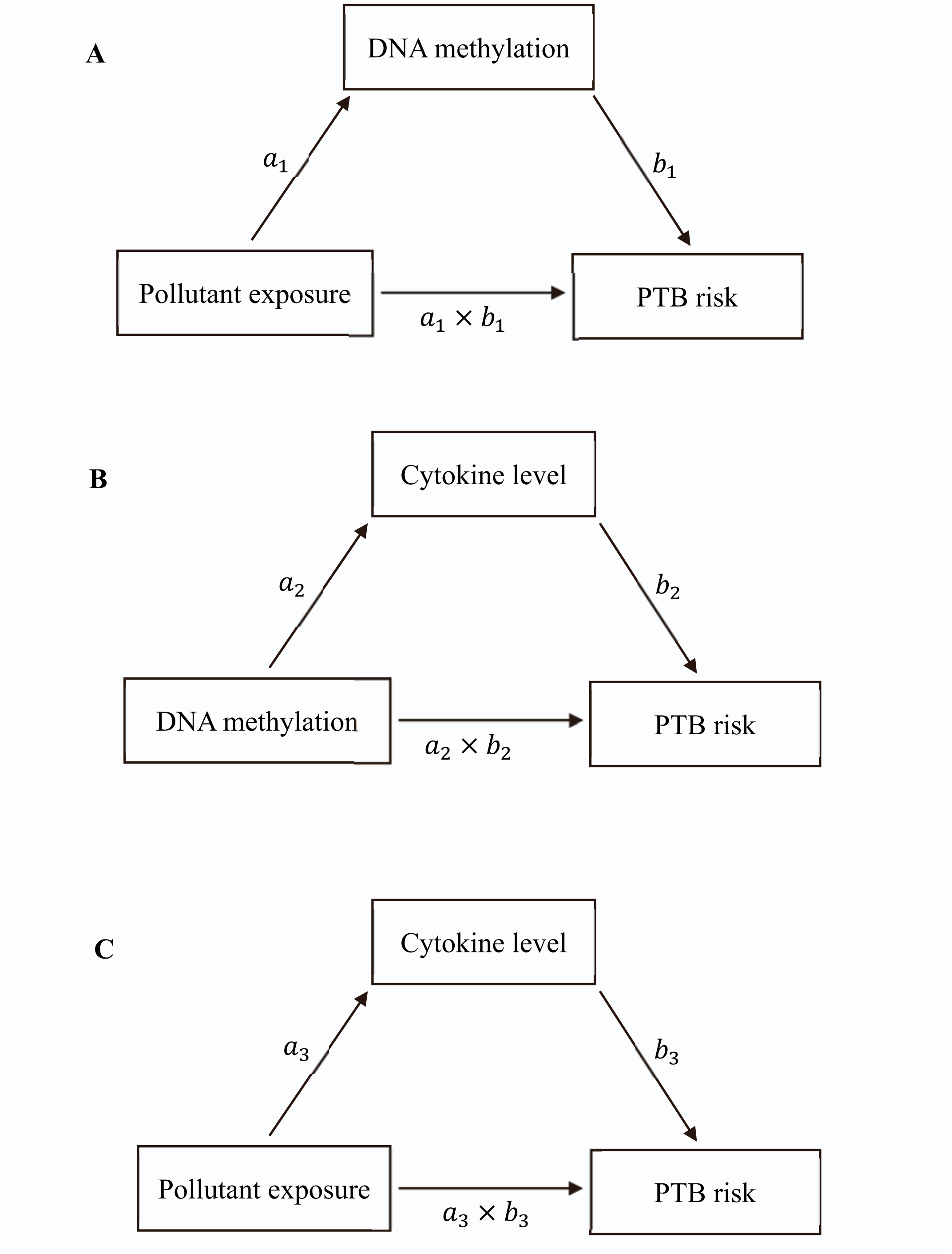

Figure S1. Models showing the associations tested in the mediation analysis, related to Table 4.

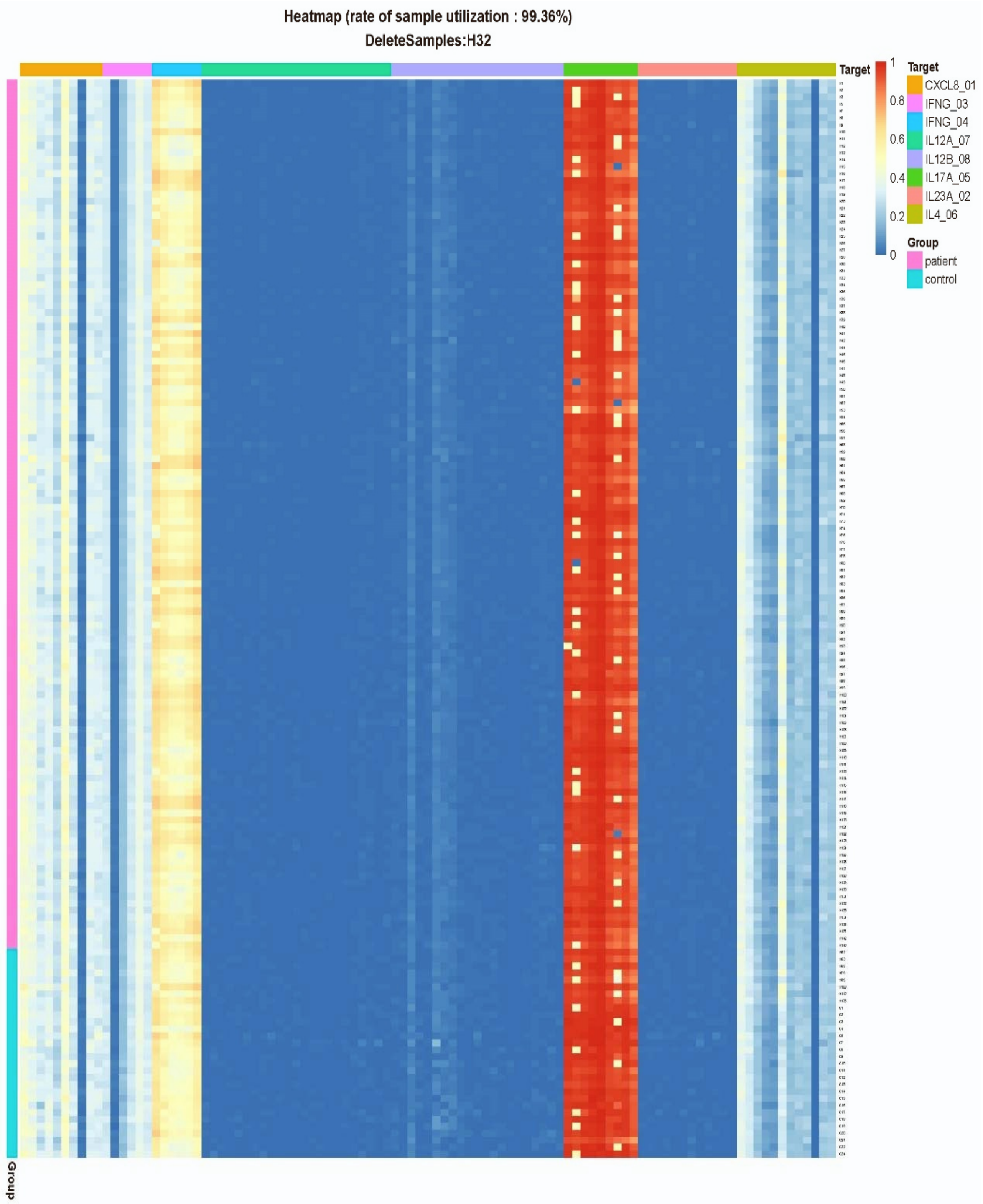

Figure S2. Cluster analysis of target gene methylation levels in pulmonary tuberculosis patients and control patients, related to Table 3 and Table 4.
